# Complete Mitochondrial Genome Sequence of *Mansonella perstans*

**DOI:** 10.1128/MRA.00490-20

**Published:** 2020-07-23

**Authors:** Matthew Chung, Jain Aluvathingal, Robin E. Bromley, Suvarna Nadendla, Fanny F. Fombad, Chi A. Kien, Narcisse V. T. Gandjui, Abdel J. Njouendou, Manuel Ritter, Lisa Sadzewicz, Luke J. Tallon, Samuel Wanji, Achim Hoerauf, Kenneth Pfarr, Julie C. Dunning Hotopp

**Affiliations:** a Institute for Genome Sciences, University of Maryland School of Medicine, Baltimore, Maryland, USA; b Department of Microbiology and Immunology, University of Maryland School of Medicine, Baltimore, Maryland, USA; c Greenebaum Cancer Center, University of Maryland, Baltimore, Maryland, USA; d Research Foundation for Tropical Diseases and the Environment, Buea, Cameroon; e Parasite and Vector Research Unit, Department of Microbiology and Parasitology, University of Buea, Buea, Cameroon; f Institute for Medical Microbiology, Immunology, and Parasitology, University Hospital Bonn, Bonn, Germany; g German Center for Infection Research, Bonn-Cologne Partner Site, Bonn, Germany; Vanderbilt University

## Abstract

The 13,647-bp complete mitochondrial genome of *Mansonella perstans* was sequenced and is syntenic to the mitochondrial genome of *Mansonella ozzardi*. Phylogenetic analysis of the mitochondrial genome is consistent with the known phylogeny of ONC5 group filarial nematodes.

## ANNOUNCEMENT

Mansonella perstans is one of three species in the *Mansonella* genus and is a causative agent of the neglected tropical disease human mansonellosis (1). Despite the prevalence of mansonellosis, no whole-genome sequences are available for *Mansonella* spp., and only the Mansonella ozzardi mitochondrial genome has been deposited in GenBank (2, 3).

Infective larvae (L3s) were obtained from *Culicoides* midges that had been kept for 12 days in the laboratory following a blood meal (1,500 microfilariae/ml) on a microfilaria-positive donor from Ediki Village, Kumba Health District, Cameroon, who had provided informed consent, as approved by the National Institutional Review Board, Yaoundé, Cameroon (protocol 2015/09/639/CE/CNERSH/SP), and the Delegation of Public Health, South West Region, Cameroon (protocol R11/MINSANTE/SWR/RDPH/PS/259/382), as described previously (3). The objectives of the study and safety procedures were explained to the volunteer, who provided signed consent and received mebendazole to cure the M. perstans infection at the study conclusion. Isolated L3s were cultured at the Department of Microbiology and Parasitology, University of Buea (Buea, Cameroon), on a confluent monolayer of monkey kidney epithelial cells in Dulbecco’s modified Eagle’s medium supplemented with 10% fetal bovine serum (4). After 50 days, viable juvenile adult worms were isolated, and DNA was extracted using a QIAamp kit with overnight incubation at 56°C. Genomic DNA was sheared using a Covaris E210 ultrasonicator, and KAPA HyperPrep libraries were constructed for two samples, with 29,320,288 and 30,552,342 paired-end 151-bp reads being generated on the Illumina HiSeq 4000 platform; reads were quality controlled with FastQC v0.11.7 (5) and trimmed with Trimmomatic v0.38 (6). Default software options were used except where otherwise noted. Sequencing reads were mapped to the M. ozzardi mitochondrial genome (GenBank accession number KX822021.1) using BWA-MEM v0.7.17 (7) with a seed length of 23, extracted, and used to assemble the M. perstans mitochondrial genome using GetOrganelle v1.6.2e (8) (with the M. ozzardi mitochondrial genome as a reference), which circularizes and trims the genome. Circularization was confirmed with Bandage v0.8.0 (9).

**FIG 1:**
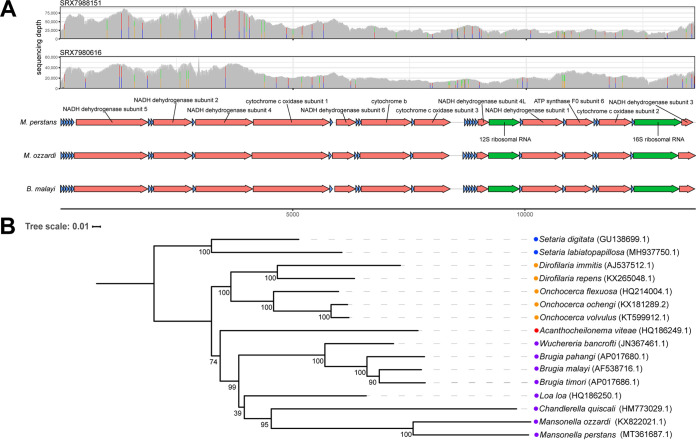
(A) The plots at the top show the sequencing depth across the M. perstans genome, as assessed from two separate libraries. Vertical colored lines on the depth tracks are indicative of positions at which a minor base call contributes >25% of the base calls at that position. Each line is colored proportionally for the different base calls at the variant position. Black and red boxes are indicative of positions at which insertions or deletions, respectively, contribute >25% of the sequencing depth at that position. Under the depth tracks is a feature comparison of the M. perstans, M. ozzardi, and B. malayi mitochondrial genomes, which shows that the two mitochondrial genomes are largely syntenic and similar in feature content, including coding sequences (red), tRNAs (blue), and rRNAs (green). (B) A maximum likelihood phylogenetic tree was generated using the M. perstans mitochondrial genome assembly and the mitochondrial genomes of 15 other filarial nematode species. GenBank accession numbers for the mitochondrial genomes are listed to the right of the species names, with colored circles denoting the ONC2 (blue), ONC3 (orange), ONC4 (red), and ONC5 (purple) clades.

The 13,619-bp complete M. perstans mitochondrial genome was assembled with a median depth of coverage of 1,115×. The genome has a G+C content of 25.9% and 85.8% sequence identity (BLASTn) to the M. ozzardi mitochondrial genome with no genomic rearrangements (Fig. 1A), as assessed using NUCmer v3.23 (10) and Artemis comparison tool v17.0.0 (11). A maximum likelihood phylogenetic tree was constructed with 15 related mitochondrial sequences (Table 1) using MAFFT v7.427 (12), IQ-TREE v1.6.2 (13) run with ModelFinder (14) and 1,000 ultrafast bootstrap replicates (15), and iTOL v5 (16). The topology is consistent with a multilocus phylogeny that places M. perstans in the ONC5 clade (17) with the agents of lymphatic filariasis and loiasis (Fig. 1B).

**TABLE 1:** GenBank accession numbers for filarial nematode mitochondrial genomes

GenBank accession no.	Species
HQ186249.1	*Acanthocheilonema viteae*
AF538716.1	*Brugia malayi*
AP017680.1	*Brugia pahangi*
AP017686.1	*Brugia timori*
HM773029.1	*Chandlerella quiscali*
AJ537512.1	*Dirofilaria immitis*
KX265048.1	*Dirofilaria repens*
HQ186250.1	*Loa loa*
KX822021.1	*Mansonella ozzardi*
MT361687.1	*Mansonella perstans*
HQ214004.1	*Onchocerca flexuosa*
KX181289.2	*Onchocerca ochengi*
KT599912.1	*Onchocerca volvulus*
GU138699.1	*Setaria digitata*
MH937750.1	*Setaria labiatopapillosa*
JN367461.1	*Wuchereria bancrofti*

The M. perstans mitochondrial genome was annotated using GeSeq v1.81 (18) with ARWEN v1.2.3 (19) and the MITOS Web server with the invertebrate genetic code (20), followed by extensive manual curation. The content of the M. perstans mitochondrial genome is largely identical to that of the M. ozzardi and Brugia malayi mitochondrial genomes, containing 12 coding sequences, 22 tRNAs, and 1 copy each of the 12S and 16S rRNA genes (Fig. 1A).

Sequencing reads from both M. perstans sequencing libraries were aligned to the M. perstans mitochondrial genome assembly using BWA-MEM v0.7.17 (21) with a seed length of 23 (7), and IGV v2.3.81 (22) and R v4.0.0 (23) were used to visualize single-nucleotide polymorphisms (SNPs) and indels (Fig. 1A). These SNPs and indels could be due to population-level differences or nuclear-mitochondrial gene transfer reads from M. perstans obfuscating the mitochondrial genome assembly, which will have to be examined in more detail in the future.

### Data availability.

The M. perstans mitochondrial genome sequence has been deposited in GenBank under the accession number MT361687. Reads mapping to the M. perstans mitochondrial genome assembly have been deposited in the SRA under the accession number SRP253836. Source code for reproducing the computational analyses described in this paper can be downloaded from https://github.com/Dunning-Hotopp-Lab/Complete-mitochondrial-genome-sequence-of-Mansonella-perstans. All code is made available under the MIT License.
